# What Features and Functions Are Desired in Telemedical Services Targeted at Polish Older Adults Delivered by Wearable Medical Devices?—Pre-COVID-19 Flashback

**DOI:** 10.3390/s20185181

**Published:** 2020-09-11

**Authors:** Eliasz Kańtoch, Anna Kańtoch

**Affiliations:** 1 AGH University of Science and Technology, 30-059 Kraków, Poland; 2 Faculty of Medicine, Department of Internal Medicine and Gerontology, Jagiellonian University Medical College, 30-688 Kraków, Poland; anna.kantoch@doctoral.uj.edu.pl

**Keywords:** wearable, gerontechnology, health monitoring system, assisted living technologies, telemedical services

## Abstract

The emerging wearable medical devices open up new opportunities for the provision of health services and promise to accelerate the development of novel telemedical services. The main objective of this study was to investigate the desirable features and applications of telemedical services for the Polish older adults delivered by wearable medical devices. The questionnaire study was conducted among 146 adult volunteers in two cohorts (C.1: <65 years vs. C.2: ≥65 years). The analysis was based on qualitative research and descriptive statistics. Comparisons were performed by Pearson’s chi-squared test. The questionnaire, which was divided into three parts (1-socio-demographic data, needs, and behaviors; 2-health status; 3-telemedicine service awareness and device concept study), consisted of 37 open, semi-open, or closed questions. Two cohorts were analyzed (C.1: *n* = 77; mean age = 32 vs. C.2: *n* = 69; mean age = 74). The performed survey showed that the majority of respondents were unaware of the telemedical services (56.8%). A total of 62.3% of C.1 and 34.8% of C.2 declared their understanding of telemedical services. The 10.3% of correct explanations regarding telemedical service were found among all study participants. The most desirable feature was the detection of life-threatening and health-threatening situations (65.2% vs. 66.2%). The findings suggest a lack of awareness of telemedical services and the opportunities offered by wearable telemedical devices.

## 1. Introduction

Wearable sensors have been successfully applied in motion analysis (e.g., physical activity recognition [1], gait assessment [2], compensatory balance reactions detection [3]), life-space mobility [4], age-related chronic diseases monitoring (e.g., neurodegenerative disease [5], anxiety [6]), and risk assessment [7]. Furtherer promising application of wearable sensors includes a comprehensive geriatric assessment when performing gait speed assessment and The Timed Up and Go (TUG) test provided by geriatricians, e.g., in assessing functional mobility and identifying patients at risk of falling; screening for the frailty syndrome using different frailty assessment scales, and screening for sarcopenia using the SARC-F questionnaire (the acronym SARC-F contains 5 domains: S- strength, A- assistance with walking, R- rising from a chair, C- climbing stairs, F- falls) [8,9,10]. Wearable sensors may address selected health care and social challenges due to the aging population and accelerate the transition to personalized digital medicine. Health insurance companies are interested in wearable fitness trackers to quantify health risks, i.e., Oscar insurance company pays $1 a day when users meet a daily step goal [11]. Telemedical and digital technologies are regarded as potential support for improving the effectiveness of healthcare services and may become part of the solution for the number of healthcare challenges, including the rapidly aging population [12]; limited access to primary care, beds in hospitals, nursing homes, specialists, and other health care services; or emergency scenarios (e.g., natural disasters, pandemics).

However, this study is, to the best of our knowledge, the first to focus on the investigation of understanding, features, and functions of telemedical services delivered by wearable sensors and desired by older adults in Poland. This knowledge is also particularly crucial for policymakers and manufacturers of medical devices as the input data for the design and development process and clinical evaluation of medical devices. The need for this novel knowledge has been highlighted in the conclusion of a recently published comprehensive—a systematic review by Stavropoulos et al. [13]. The authors concluded that future work might focus on the aspect of IoT (Internet of things) for eldercare like human needs or hardware and software features. This study faced this challenge in the presented scope.

The main objective of this study was to investigate the knowledge, opinion, approach, attitude, and the perspective of telemedical services among adults that may use such telemedical services (the geriatric population and their relatives). Participants evaluated the features and functions of prototype wearable health telemonitoring systems (e.g., size, ergonomics, way of using, features, functions) and expressed their opinion and interests of using this system in everyday life. The novelty of this study was the following: a unique set of questions prepared based on experience in designing and building telemedicine medical systems based on medical wearable sensors supported by extensive explanations of the existing wearable telemedical system prototype; focusing on the user’s medical needs; study population, which consists of two selected cohorts (users of the system vs. users of telemedical services); analysis of the system feasibility and interdisciplinary expertise from a clinical and technological point of view; original findings in the form of the list of features and functions of desired wearable medical devices and remarks concerning applications and analysis of wearable medical device certification process from a regulatory perspective. Last but not least, the presented results can reflect the knowledge of older people about the possibilities of wearable sensors and telemedicine before the COVID-19 (coronavirus disease 2019) pandemic and provide guidelines for policymakers and stakeholders involved in developing wearable telemedical services for seniors. 

The paper is structured as follows. Section 2 presents materials and methods. Section 3 presents the original results. Section 4 presents related work and discusses the outcomes of the study. Section 5 presents guidelines for policymakers and stakeholders and regulatory perspective in the area of wearable telemedical devices, and Section 6 concludes the paper.

## 2. Materials and Methods

### 2.1. Participants

The presented survey was carried out among 146 subjects divided into two age groups (cohort 1: <65 years vs. cohort 2: ≥65 years old) in Poland. The study was performed among adult volunteers in academic, domestic, social, and Internet settings. The main inclusion criteria were signed informed consent. Subjects with dementia or cognitive impairment were excluded from the study.

### 2.2. Objective

The main objective of the study was to investigate the features and functions of telemedical services delivered by wearable sensors and desired by older adults in Poland. In addition, the study aimed to assess the understanding, knowledge, opinion, approach, attitude, and perspective of telemedical services among adults who may benefit from such a telemedical service (geriatric population and their relatives).

### 2.3. Procedure

The survey could be completed online or by hand. The information about the study was officially put on many websites with similar studies. The link to the survey was also sent via e-mail. An online survey could only be completed if the participant filled in the full form. The hand version of the questionnaire, which was easier to complete by the elderly people, was completed by qualified and trained pollsters and next entered the database. Each study participant was asked to fill out all the survey fields. The questionnaire consisted of 37 questions that were open, semi-open, or closed with the possibility of single or multiple choice. The questions included in the survey were structured in such a way as to be simple, understandable, and unambiguous, especially for the elderly.

The unique questionnaire was divided into three parts. The first part concerned the socio-demographic data and familiar status, needs, and behaviors; the second part consisted of questions about health status, their contact with their general practitioner (GP), and finally, in the third part, questions about the awareness of the telemedical services and device concept study were included. The analysis was based on qualitative research and descriptive statistics. The participants were divided into two age groups. We carried out analysis by using a Pearson’s chi-squared test in order to evaluate whether there was a significant association between age group and understanding of telemedical services as well as a willingness to use such services. All statistical analyses were performed in *Statistica 13*. *p*-values of ≤0.05 were considered statistically significant. 

The survey also provided extensive explanations of telemedical systems concepts to facilitate understanding. The explanations were based on the developed patented wearable-based telemedical system for the Telemedical Assessment of Cardiovascular Risk demonstrator [14]. The system integrates configurable wearable sensors that track patient motion and vital signs. The server analyzes the personalized health record in real-time and highlights abnormal or unusual physiological data patterns, and informs family or emergency services. The algorithms may be configured to support the recognition of defined medical conditions, including heart failure, arrhythmia, falls, fever or low body temperature, irregular heartbeat, stress and anxiety, behavior change, and others. The general goal of the implemented health telemedical system is to provide the feeling of safety to senior citizens living alone and to facilitate the detection of emergencies that require medical intervention. The identified use-case scenario included system application in geriatric hospital departments and among the institutionalized population, especially among nursing home residents. The residents of long-term care facilities are a very specific population, often disabled, frail, malnourished, and with multimorbidity, including dementia or cardiovascular disease [15], so this method may help prevent and detect many adverse events and emergencies that may be associated with increased mortality, such as falls and injuries or fever that can be so critical during health crisis (e.g., the COVID-19 pandemic), especially during night shifts when the number of medical personnel is reduced.

## 3. Results

### General Characteristics of the Population

We analyzed two cohorts: cohort 1 (*n* = 77; mean age = 32 years old) and cohort 2 (*n* = 69; mean age = 74 years old). Most of the respondents were women 71.7% (71.4% vs. 72.5%), and they were coming from the Silesia Region (57.1%) or the Lesser Poland (20.6%). Furthermore, they were living in the countryside (37.8%) or the city with over 200,000 citizens (25.5%). They were living either in their apartments (61.0%) or houses (39.0%) with their family—mostly with their spouse (37.7%) or children (35.0%). A comparison of results between the two cohorts is presented in Table 1.

The performed survey showed that most of the study participants were unaware of the telemedical services (56.8%). A total of 62.3% of cohort 1 and 34.8% of cohort 2 declared an understanding of telemedical services. However, subjects who were familiar with this concept could not provide the correct explanation and had problems with defining this service and giving an appropriate example when being asked in the following open question. There were only 10.3% of correct explanations of telemedical service among all study participants. What is more, respondents (94.8% of cohort 1 vs. 95.7% of cohort 2) admitted that they had never used the telemedical service before. The assessment of the suitability of the properties of such a device was provided in the questionnaire on a scale of 1–5 (5-more useful). The highest rank was marked to the following properties (from the highest-ranking):

**A. Answers of cohort 1:**

- Detection of life-threatening and health-threatening situations (66.2%)
- Comfortable to wear on a daily basis (54.5%)
- Detection of abnormal functioning of the body, including illness (49.4%)
- Read health information from the device by a healthcare professional, such as a doctor (48.1%)
- Automatically informing selected individuals or institutions of danger (healthcare professionals, such as the attending physician) (45.5%)
- Self-read health information from the device (45.5%)
- Replacement of medical visits (45.5%)

**B. Answers of cohort 2:**

- Detection of life-threatening and health-threatening situations (65.2%)
- Detection of abnormal functioning of the body (56.5%)
- Automatically informing selected individuals or institutions of danger (healthcare professionals, such as the attending physician) (53.6%)
- Continuous monitoring of health status (52.2%)
- Read health information from the device by a healthcare professional, such as a doctor (49.3%)

It was observed that both the younger and older population ranked the three most essential device properties in the same order. However, the properties of the device were mostly marked as useful (4) or more useful (5). The majority of the participants found those properties useful, and they were willing to use that device in their daily life. Younger respondents were more indifferent (3) (19.1%) than the older (14.3%) in defining the device properties. Both age groups (cohort 1: 9.1% vs. cohort 2: 21.7%) were interested in using the solution characterized by such features, as described. Paradoxically, younger people were less interested in using that device and declared that they would use it if they became chronically ill or needed it. The older population was skeptical about using the device because of fear of large service costs, so the elderly reported preferably contacting their doctor directly rather than using telemedicine. They explained their choice by poor knowledge about technological possibilities. Younger participants declared that they did not need to use such a device at present because of the lack of health problems, and they were not interested in being monitored all the time. All study participants answered that they were interested in sending the message about the worsening of health condition to their general practitioner (50.0%), family (46.6%), or the emergency medical services (15.1%). They highlighted the significance of this practice concerning the sense of security, providing quick help in a state of danger or physical comfort. 

Respondents were also asked to imagine the most comfortable way of wearing the equipment of telemedical service—they indicated different ways of wearing the concept of telemedical devices like the pocket, purse, watch, necklace, bracelet, wristband, belt, smartphone, or app in their smartphone. Moreover, they were asked to choose the most suitable and comfortable size of the concept device between matchbox size, smartphone size, or bar of chocolate size. As a result, the matchbox size was chosen as the most appropriate (71.2%), while the bar of chocolate size was indicated as too large (82.9%) to wear. The technological skills were also assessed among respondents. Most of them were able to use the computer (79.5%), smartphone (69.2%), or the Internet (80.1%), but as far as life-measuring devices are concerned, more than half were using blood pressure gauge, while the pulse meter was used only in 17.1%. 

## 4. Related Work and Discussion

### 4.1. Wearable Technologies

Rawassizadeh et al. [16] recognized the potential of smartwatches for the development of mHealth technologies and their increasing role in bio-measurements. He listed battery life and cost as the most crucial success factors for smartwatch acceptance as well as maintaining privacy. The application of wearable technologies in health care was highlighted in previous studies. Pantelopoulos et al. [17] recognized the potential of wearable sensor-based systems for health monitoring to revolutionize healthcare by providing unobtrusive personal health monitoring and to enable early detection and better understanding and self-management of chronic diseases. However, he highlighted that there are a lot of challenges and issues that need to be resolved for wearable systems to become accepted by patients as reliable, multifunctional, easy-to-use, and minimally obtrusive. Majumder et al. [18] presented a survey on wearable physiological parameters and activity monitoring systems. He identified the primary purpose of a wearable health monitoring system to allow people to lead independent and active lives in a home environment while ensuring non-invasive surveillance of their health. He recognized that wearable sensors and actuators, coupled with information and communication technologies, might be useful in cost-effective remote healthcare services. The performed study results were comparable to the findings of Stavropoulos et al. presented in the comprehensive, systematic review of studies in IoT wearables and devices for eldercare [13]. They identified four major health focus areas: Alzheimer’s disease, Parkinson’s disease, dementia, and fall detection. The main aim of the reviewed studies was health monitoring, fall detection, movement analysis, gait analysis, and disease assessment. They examined the following criteria: healthcare impact, cost-effectiveness, acceptance-usability, and infrastructure. The authors found that telemedical application was present in the only one review study that confirmed the initial hypothesis of low awareness of wearable-based telemedical services. The authors also found that patients involved in the study provided positive feedback for some technologies.

### 4.2. Telemedicine

According to WHO statistics, the number of people aged 60 years and over will reach 22% (2 billion) of the population in 2050 in comparison to 11% (605 million) in 2000 [19]. As a result, this will increase the demand for health care services. At present, about 44% of WHO Member States report having less than one physician per 1000 population, and this number is unlikely to increase in the future [19]. Telemedicine enables the remote exchange of data between a patient and healthcare professionals to facilitate diagnosis, monitoring, and management of long-term conditions [20]. There is evidence that telemedical systems and assistive living technologies can improve the quality of a patient’s life. The largest randomized control trial of telemedicine involving 6191 patients and 238 general practices study showed that telehealth could reduce the mortality rates by 45%, emergency admissions by 20%, and reduction in bed days by 14% [21]. However, despite many viable solutions, wearable telemedical services are not widely adopted in clinical practice.

The growing number of different smart electronic devices is observed in the environment of older adults. The older adults often use special phones equipped with a panic button that sends the message—“call for help”. To make telemedical services more accessible for the elderly, we need to better understand the elderly needs, listen to them without prejudice, and integrate the service into everyday objects like intelligent health devices that will monitor patient health without limiting patient movement. The performed study showed that both the population of the elderly and younger people had problems with not only defining the telemedicine but also with giving the example of this kind of service. A similar opinion of older adults was presented by Cimperman et al. [22]. Only a few study participants pointed out that they used teleconsultation with a physician by phone. According to our survey respondents, telemedical services should provide a sense of security, comfort, quick help, and should not be the replacement of the traditional visit to the doctor. 

Merrell et al. suggested that telemedicine should be integrated with physician’s work in a way that makes it possible to improve [23]. It could be an option chosen, especially among the elderly with multimorbidity, to monitor their health status and to remind them about taking medications rather than the necessity to adopt. The systematic review of virtual visits was presented by Husebø et al. [24]. Another aspect could be the fact that older people still prefer face-to-face visits what makes newer models of providing e-health very challenging to adopt among this age group [25]. The respondents highlighted that this idea should be developed, and doctors should be more interested in new technologies. Their opinion could be confirmed by the study performed in Iran in 2013, which showed that clinicians had limited knowledge about telemedicine [26]. 

We found that the concept of the telemedical device with identified properties is desirable, and the study participants indicated the list of functionality for such a telemedical device. The older population of study participants was willing to use the telemedical device on their own, while the younger respondents were interested in buying this service for their older relatives with chronic diseases. We shall encourage people to be more open to new technological solutions by providing the necessary information and training. Many previous studies have proved that such training could help the elderly to increase the intention to use technology and build their self-confidence [27,28,29]. On the other hand, there are some threats—a young population of respondents indicated that the sense of privacy was crucial, and this kind of device should not have been the mechanism of surveillance, breaking privacy, or breaching personal data. This problematic aspect was analyzed and presented in the systematic review of telemedicine security [30]. Cognitive, visual, or hearing impairments could also be regarded as a limitation of the use of the telemedical device. What is more, this kind of device should not be regarded as being stigmatizing or embarrassing [31,32,33]. There is also another danger of this approach—telemedical service could deepen the social isolation of the older adults, and their relatives may visit them less often because they are provided by telemedical services [32]. 

The presented results are retrospection of knowledge about telemedical devices in two age groups in the pre-pandemic period when telemedical services and the possibilities offered by telemedicine were unknown. It cannot be ruled out that greater awareness of telemedical services and monitoring of chronic diseases among the elderly before the outbreak of the epidemic would result in lower mortality in this age group during the COVID-19 pandemic in Poland. Conversely, during the COVID-19 pandemic, telemedical services gained traction all over the world, and more and more people, including the elderly, were interested in using such solutions in everyday life to feel safer. 

### 4.3. From a Clinical Point of View

Most older adults have more than one chronic disease. Multimorbidity and polypharmacy are very common in the geriatric population. However, they are associated with an increased risk of mortality, adverse drug events, disability, poor functional status, and low quality of life [34]. The most common chronic diseases in older adults are cardiovascular diseases (e.g., hypertension, chronic heart failure), dementia (e.g., Alzheimer’s disease), diabetes mellitus, and cancer. As far as the physical function is concerned, disability and frailty syndrome are also widespread in this population, especially among nursing home residents [35,36]. Understanding the needs and opinions of older adults with chronic diseases seems to be crucial for the provision of the best telemedical services.

#### 4.3.1. Hypertension

The benefits of using telehealth among hypertensive older adults were demonstrated in 2014 by Czaja et al. In this study, the telemedical system installed in the participants’ homes sent data on the participants’ blood pressure and weight to the server. Most of the respondents (92%) admitted that using the device is easy. Moreover, the application of the system resulted in the improvement of reported behaviors in the field of health management [37].

#### 4.3.2. Chronic Heart Failure

In 2018, Clark investigated the effectiveness and satisfaction of using telehealth in elderly patients with heart failure. The use of telemonitoring and structured phone calls in this population was associated with a reduced risk of all-cause mortality and heart failure-related hospitalizations. However, technologies, including videophones or interactive voice responses, have not been as effective. What is more, the level of satisfaction of the respondents with the telehealth capabilities ranged from 76% to 97%. These results proved that older adults could quickly adapt to this type of telemedical service (adherence to recommendations was 55.1–98.5%) if the benefits are related to their health safety and reduced risk of hospitalization due to exacerbation of their chronic disease—heart failure [38].

#### 4.3.3. Diabetes Mellitus

A meta-analysis of 42 randomized, controlled trials conducted by Tchero et al. showed that telemedicine intervention is more effective in managing diabetes in elderly patients than ordinary care [39].

#### 4.3.4. Dementia

Population aging is associated with an increase in the number of people diagnosed with dementia. Telemedicine and smart technologies can support caregivers and people with dementia at home. Robot technologies have been developed to support necessary activities in daily living and positively associated with increasing the quality of life of older people with cognitive impairments [40]. 

#### 4.3.5. Frailty Syndrome

Frailty syndrome among older individuals could be associated with several adverse events, including increased mortality and risk of hospitalization. In this case, telemedicine could play a critical role in identifying pre-frailty conditions through daily home monitoring with a pendant sensor, as shown in 2018 by Razjouyan et al. [41].

#### 4.3.6. Mobility

Application of wearable sensors for older adults was previously studied, e.g., by Ho et al., who developed a hybrid mobility tracking system that tracks the mobility of older people outdoors, which can help understand older adults’ travel and activity patterns outside the home [4]. What is more, Jansen et al. demonstrated the beneficial effects of physical activity intervention on life-space utilization as assessed by an objective, sensor-based system in nursing home residents [42]. Gerhardy et al. proposed the Timed Up and Go analysis for quick screening for underlying sensory deficits related to clinically relevant impairments in functional performances [43].

The study had some limitations. The study did not provide the opinion of the elderly with dementia or with cognitive impairment, whose opinion may be crucial in providing health assistance among the elderly population. This group can most benefit from telemedical services to improve their sense of safety at home, allowing them to live more independently. Furthermore, the presented qualitative research should be regarded as exploratory rather than confirmatory, which is also a limitation of the findings. Telemedical healthcare shall provide the maximum of human independence, usefulness, and self-determination. Moreover, it should be individualized, and it should meet all individual’s requirements by the caregiver and doctor. The sense of privacy should always be respected. Telemedical service is the chance for a sense of safety among the elderly, but this kind of system should be the support rather than a replacement for a physician. Furthermore, it should be affordable, intuitive, and easy to use as a smartwatch. 

## 5. Guidelines for Policymakers and the Designers of the Wearable Telemedical Systems

This study aimed to identify the desired features and functions of wearable telemedical systems. Findings from the conducted study suggest that the most desired function of the wearable telemedical systems are:

- Detection of life-threatening and health-threatening situations
- Providing quantified medical data, e.g., heart rate, physical rehabilitation recognition
- Automatically informing selected individuals or institutions of danger (healthcare professionals, such as the attending physician)

However, the critical requirement for application is the use-case scenarios:

- Comfortable to wear on a daily basis and not limiting patient movement
- Low energy consumption (maintenance-free, ideally batteryless operation)
- Accurate medical parameters measurements (clinical value)

The most desired applications were:

- Tracking quality and quantity of physical rehabilitation
- Recognition of physical activity patterns
- Detection of falls or injuries
- Indoor and outdoor positioning
- Tracking parameters that are used in the clinical assessment tools to evaluate the patient’s clinical condition or predict the risk or prognosis

Our findings suggest that tracking and providing feedback on medical parameters is important for the majority of study participants. The most desired monitoring parameters were motion pattern, heart rate, ECG, oxygen saturation, body temperature, skin conductance (affective reactions), glucose level, blood pressure, noise, and user location indoors and outdoors (GPS).

We found that the significant fears and barriers for the adoption of wearable telemedical systems were:

- Cost of the service
- Difficulty to use and configure
- Interoperability
- Mindset barrier (preference of seeing doctor personally)
- Poor knowledge about the potential benefits of telemedical services
- Complex certification process

European manufacturers of wearable medical devices shall apply for specific conformity assessment procedures with the notified body whom they must involve in the certification process. In the European Union, medical devices based on wearable sensors shall be certified in accordance with the requirements of Regulation (EU) 2017/745 of the European Parliament and of the Council of 5 April 2017 on medical devices, amending Directive 2001/83/EC, Regulation (EC) No 178/2002 and Regulation (EC) No 1223/2009, and repealing Council Directives 90/385/EEC and 93/42/EEC to ensure safety and declared performance [44]. The critical certification requirements include defined medical purpose, clinical investigation in case of the lack of sufficient clinical data to support the claims on safety and performance of a medical device. Further requirements include compliance with harmonized standards under Union harmonization legislation. The essential standards in discussed scope are EN 60601-1-1 Medical electrical equipment—Part 1-1: General requirements for safety—Collateral standard: Safety requirements for medical electrical systems; EN 60601-1-2 Medical electrical equipment—Part 1-2: General requirements for basic safety and essential performance—Collateral Standard: Electromagnetic disturbances—Requirements and tests; EN 60601-2-27 Medical electrical equipment—Part 2-27: Particular requirements for the safety, including essential performance, of electrocardiographic monitoring equipment; EN 62,304 Medical device software—Software life-cycle processes; EN ISO 14,971 Medical devices—Application of risk management to medical devices; EN 62,366 Medical devices—Application of usability engineering to medical devices. The above requirements shall be analyzed during the medical device development process. 

## 6. Conclusions and Future Works

This study is, to the best of our knowledge, the first to present desirable features and functions of telemedical services targeted at older adults delivered by wearable medical devices. The most desired feature of a wearable-based telemedical system was the detection of life-threatening and health-threatening situations. We found that inadequate knowledge of telemedical services was present among both young and older people, so the popularization of telemedical services is needed to broaden the knowledge about telemedicine and accelerate the development of telemedical services. It could be achieved by organizing conferences, meetings, talks, presentations, or advertisements. Furthermore, decision-makers should consider introducing measures to facilitate adoption and raise awareness of telemedical services. Future works will focus on comparing and contrasting pre- and post-pandemic period. We will analyze the privacy and security of the medical data harvested from wearable systems. The regulatory aspects and certification requirements for wearable medical devices will also be investigated.

## 7. Patents

Kańtoch, E.; Kańtoch A. Patent granted number 418874. Title: “The method of signal acquisition, sensor sticker and control-measurement system” (granted 2019).

## Figures and Tables

**Table 1:** A comparison of results between the two cohorts (cohort 1 vs. cohort 2).

Variable		Cohort 1 (*n* = 77 )	Cohort 2 (*n* = 69)	*p*-Value
Female sex (%)		71.4	72.5	
Declare the understanding of telemedical service (%)		62.3	34.8	0.05
Have used telemedical service before (%)		5.2	4.3	
Suitability of the following properties on a scale 1–5 (5-more useful)	Scale (1–5)			
A. Continuous monitoring of health status (%)				
	1	9.1	4.3	
	2	3.9	7.2	
	3	24.7	13.0	
	4	29.9	23.2	
	5	32.5	52.2	
B. Health monitoring at the time of your choice (%)				
	1	6.5	4.3	
	2	6.5	10.1	
	3	26.0	13.0	
	4	29.9	30.4	
	5	31.2	42.0	
C. Detection of life-threatening and health-threatening situations (%)				
	1	2.6	2.9	
	2	0	1.4	
	3	11.7	8.7	
	4	19.5	21.7	
	5	66.2	65.2	
D. Detection of abnormal functioning of the body (%)				
	1	5.2	5.8	
	2	1.3	1.4	
	3	16.9	10.1	
	4	27.3	26.1	
	5	49.4	56.5	
E. Self-read health information from the device (%)				
	1	7.8	5.8	
	2	1.3	7.2	
	3	24.7	17.4	
	4	20.8	27.5	
	5	45.5	42.0	
F. Read health information from the device by a healthcare professional such as a doctor (%)				
	1	7.8	4.3	
	2	3.9	8.7	
	3	18.2	13.0	
	4	22.1	24.6	
	5	48.1	49.3	
G. Automatically informing selected individuals or institutions of danger (healthcare professionals, such as the attending physician) (%)				
	1	5.2	2.9	
	2	2.6	4.3	
	3	18.2	14.5	
	4	28.6	24.6	
	5	45.5	53.6	
H. Comfortable to wear on a daily basis (%)				
	1	6.5	5.8	
	2	3.9	5.8	
	3	15.6	18.8	
	4	19.5	26.1	
	5	54.5	43.5	
I. Replacement of medical visits (%)				
	1	10.4	4.3	
	2	7.8	11.6	
	3	15.6	10.1	
	4	20.8	26.1	
	5	45.5	47.8	
J. Replacement of hospitalization (%)				
	1	10.4	7.2	
	2	11.7	7.2	
	3	19.5	13.0	
	4	15.6	24.6	
	5	42.9	47.8	
K. Use health information of many patients monitored to better protect the health and life of other patients and the development of medicine (%)				
	1	3.9	4.3	
	2	6.5	2.9	
	3	19.5	20.3	
	4	22.1	29.0	
	5	48.1	43.5	
				
Interest in using a solution characterized by described features (%)		9.1	21.7	0.05
Declare that this device could change their lives (%)		50.6	71.0	0.01
